# Evidence of morphine like substance and μ-opioid receptor expression in *Toxacara canis* (Nematoda: Ascaridae)

**Published:** 2016-12-15

**Authors:** Mostafa Golabi, Soraya Naem, Mehdi Imani, Nowruz Dalirezh

**Affiliations:** 1 Department of Pathobiology, Faculty of Veterinary Medicine, Urmia University, Urmia, Iran; 2 Department of Basic Sciences, Faculty of Veterinary Medicine, Urmia University, Urmia, Iran; 3 Department of Microbiology, Faculty of Veterinary Medicine, Urmia University, Urmia, Iran

**Keywords:** HPLC, Immune modulation, Morphine-like substance, RT-PCR, *Toxacara canis*

## Abstract

*Toxocara canis* (Nematoda: Ascaridae) is an intestinal nematode parasite of dogs, which can also cause disease in humans. Transmission to humans usually occurs because of direct contact with *T. canis* eggs present in soil contaminated with the feces of infected dogs. This nematode has extraordinary abilities to survive for many years in different tissues of vertebrates, and develop to maturity in the intestinal tract of its definitive host. Survival of parasitic nematodes within a host requires immune evasion using complicated pathways. Morphine-like substance, as well as opioids, which are known as down regulating agents, can modulate both innate and acquired immune responses, and let the parasite survives in their hosts. In the present study, we aimed to find evidences of morphine-like substance and µ-opiate receptor expression in *T. canis*, using high performance liquid chromatography (HPLC) and reverse transcription polymerase chain reaction (RT-PCR). The results indicated that *T. canis* produced morphine-like substances at the level of 2.31± 0.26 ng g^-1^ wet weight, and expressed µ-opiate receptor as in expected size of 441 bp. According to our findings, it was concluded that *T. canis*, benefits using morphine-like substance to modulate host immunity.

## Introduction

Successful parasitism depends on host and parasite interaction, specifically between host’s immune system and parasite’s ability to create a permissive micro-environment *in situ*.^1^ It is estimated that over two billion people are currently infected with helminthes worldwide,^2^ however, a large percent of these infections are asymptomatic and suggest these worms may down-regulate host immune response against them via production of common signaling molecules.^3^^,^^4^ Opioids substance like morphine generally known as a down regulating agent which can modulate both innate and acquired immune responses and consequently cause resistance of infectious agent in host bony.^5^^,^^6^ These findings supported by this fact that in several mammalian tissue include brain and adrenal gland, morphine is detected.^1^ For example it has been shown that free-living and parasitic invertebrates produce opioid peptide precursors like prodynorphin, proopiomelanocortin and proenkephalin and these peptide show a high sequence identity with mammalian counterparts.^1^ In this context presence of opiate substance are documented from invertebrates like *Mytilus edulis*, *Mytilus galloprovincialis* and *Planorbariuscorneus*.^7^^,^^8^ In leech, *Hirudo medicinalis* like human, morphine can act as an immune inhibitor agent via up-regulation of angiotensin converting enzyme.^7^

*Toxocara canis* is a cosmopolitan and zoonotic parasite that occurs in small intestine of dog and fox with complex life cycle. According to the age of the host, transmission may involve prenatal and colostral transmission, direct transmission or paratenic host transmission.^9^In female dogs, hypobiotic larva are formed which cause heavy infection in pups during parturition or lactation.^10^ Ingestion of embryonated eggs in human cause significance clinical disease like visceral larvae migrans, ocular larvae migrans and covert toxocariasis.^11^

In mild to moderate infection, during pulmonary phase of larvae migration, usually clinical signs are non-specific. In the intestine, adult worms cause pot-belly, failure to thrive and occasional diarrhea. In heavy infections pulmonary damage, coughing, increased respiratory rate and foamy nasal discharge are seen and pups infected transplacentally may die within a few days after birth.^12^ Increase of IgE levels, eosinophilia and induction of Th-2 response which make the production of cytokines like IL-4, IL-5 and IL-13 have been seen in infected animals.^13^

According to the previous findings about morphine like substance in *Mytilus,*^14^ and *Ascaris suum*,^1^ and given that fact the *T. canis* can down regulate immune system of hosts and survive for many years in host tissue we hypothesized that it might be via a morphine like substance by which *T. canis* moderates or compromises immune responses. In this study we used the high performance liquid chromatography (HPLC) method to evaluate the production of morphine and furthermore reverse transcription polymerase chain reaction (RT-PCR) technique for expression of μ opioid receptor in *T. canis. *

## Materials and Methods

**Sample collection. **Adult *T. canis *were obtained from animal shelter of Urmia, West Azerbaijan, Iran. For this propose a newly received pups were treated by mebendazole 1g 50 kg^-1^ for five days (Tolide Darouhai Dami Iran Co. Tehran, Iran), followed by single administration of praziquantel 50 mg kg^-1 ^(Tolide Darouhai Dami Iran). Excreted worms were washed with posphate-buffered saline (PBS), and transferred to Ascaris Ringer Solution, ^15^ for HPLC analysis for five days. For RT-PCR, worms were transferred to liquid nitrogen.

**Sample preparation for HPLC. **Washed *T. canis* samples were crushed and 0.1 gram was then homogenized in 1N HCl (1 mL per 0.1 g). Adding 5 mL extraction solution (chloroform/isopropanol 9:1), samples were left for 5 min and then centrifuged twice at 3,500 rpm for 15 min. The supernatant was transferred to a new tube and centrifuged in 9,000 rpm for 15 min.

For solid-phase extraction of samples Sep-Pak plus C-18 cartridges (Chromabond, Duren, Germany) were used. First, all cartridges were activated with methanol and water then washed with phosphate buffer (pH = 6). Then, samples were loaded on cartridges and washed with phosphate buffer (pH = 4.5) and methanol, respectively. Morphine elution was performed by adding dichloro-methane/isopropyl/ethyl acetate solution (4:12:18) to the cartridges. Eluted samples were dried in 40 ˚C, 24 hr and dissolved in HPLC mobile phase prior to injection.

**HPLC condition. **The high performance liquid chromatography (hplc) analyses were performed with Knauer K-1001 pump and an Eorospher 100-5 C18 column (Knauer GmbH, Berlin, Germany). Compounds separation was monitored using a UV detector, previously described by Goumon, *et al*.^1^ Samples were eluted with a mobile phase of 90:10 (v/v) water- acetonitrile containing 0.012 M phosphate buffer (pH = 4) with a flow rate of 0.6 mL min^-1^. The column eluent was monitored at 230 nm.

**Isolation of RNA. **Isolation of RNA from *T. canis* and leech ganglia as positive control (Laurent *et al*.)^7^ was performed according to Intron RNA extraction kit instruction (Tech Dragon Lit., South Korea). Briefly, 50 mg of samples homogenized with 500 μL lysis buffer and stored in room temperature for 3 hr, then 700 μL binding buffer was added and vortexed (Genius 3; IKA-Werke, Staufen, Germany). Samples were transferred to spin columns and centrifuged at 10,000 rpm for 1 min. In the next step, 500 μL washing buffer A was added and centrifuged (10,000 rpm for 1 min) then 500 μL washing buffer B was added and centrifuged at 10,000 rpm for 1 min. After centrifuge at 10,000 rpm for 2 min, columns were transferred to new tube and 50 μL elution buffer was added, maintained in room temperature for 1 min and centrifuged at 10,000 rpm for 1 min. Collected RNA immediately was used for cDNA synthesis.^16^^,^^17^ For protection of RNA, all isolation steps were done on ice box. Purity of extracted RNA from worms were measured by 260/280 and 260/230 absorbance ratio using Nanodrop 2000c spectrophotometer (Thermo Scientific, Waltham, USA).

**cDNA synthesis and RT-PCR. **First-strand cDNA synthesis and RT-PCR were performed using One step RT-PCR kit (Qiagen, Hilden, Germany). For this propose, 5 μL of RNA was mixed with 20 μL master mix, forward and reverse primer, 1 μL reverse transcriptase-enzyme and distilled water. Tubes stored in 50 ˚C for 30 min for synthesis of cDNA and RT process. To inactivate Reverse Transcriptase process, tube transferred to 95 ˚C for 15 min. The PCR reaction consist of an initial denaturation at 95 ˚C for 5 min and 40 cycles at 95 ˚C for 1 min, 59 ˚C for 1 min, 72 ˚C for 1 min and final extension step at 72 ˚C for 10 min. The PCR products were analyzed on a 1.5% agarose gel (Sigma-Aldrich, St. Louis, USA) containing SYBR Green (CinnaGen, Tehran, Iran). The μ-opioid receptors specific primers used in PCR reaction amplifying a 441-bp fragment. Forward primer was 5'-GGTAGTGGGAAAACCTGCTGAAGATCTGTG -3' and reverse primer was 5'-GGTCTCTAGTGTTC TGACGAATTCGAGTGG-3'.^1^

## Results

**Fig. 1:**
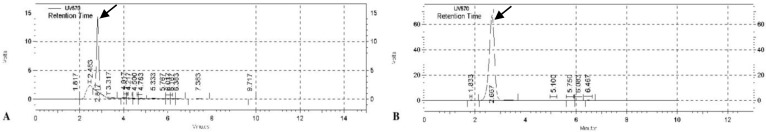
Reverse phase high performance liquid chromatography (RP-HPLC) chromatogram of morphine from *Toxocara canis*. **A)*** T. canis *extract, **B)** Standard morphine. Running conditions: Flow rate of 0.6 mL min^-1^. The column eluent was monitored at 230 nm. Mobile phase: 90:10 (v/v) water-acetonitrile containing 0.012 M phosphate buffer (pH = 4). The arrows indicate the morphine peaks in chromatogram

 **Detection of morphine like substance by HPLC. **To determine opioid alkaloid morphine utilized by *T. canis*, reversed phase HPLC was used. The chromatogram of morphine in *T. canis* extract is shown in Fig. 1A and the morphine standard chromatogram is shown in Fig. 1B.

For determination of morphine concentration, the area under sample peak which is proportional to total concentration of the morphine in the original sample was compared to the injected standard morphine. Therefore, a calibration curve was prepared by plotting peak area as a function of concentration for a series of standards. According to our results, the average concentration of morphine in *T. canis *was 2.31 ± 0.26 ng g^-1^ wet weight. 

**Fig. 2:**
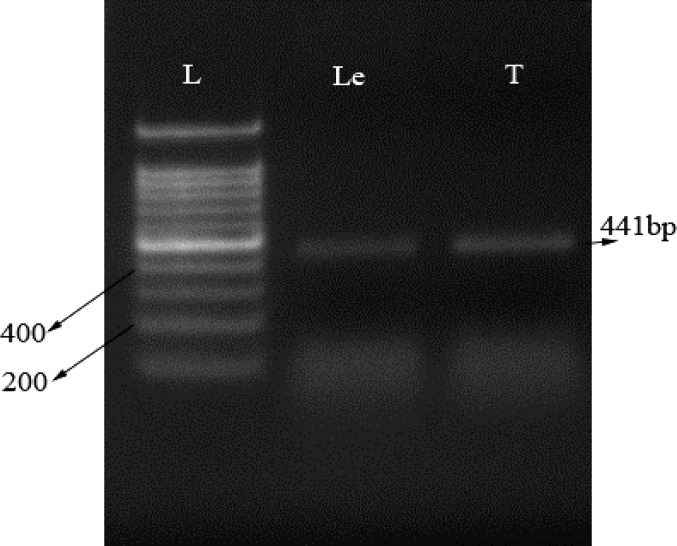
Identification of μ-opioid receptor. Total RNA were isolated, RT-PCR amplified and run on 1.5% Agarose gel. **Lane L:** Ladder; **Lane T:** μ-opioid receptor transcript in *Toxocara canis *(expected size: 441bp); **Lane Le:** μ-opioid receptor transcript in leech ganglia

**µ-opioid expression by RT-PCR. **The value for the 260/280 and 260/230 were 2.17 and 1.85 respectively, which is indicative of high quality RNA isolation. The RT-PCR used to amplify the μ-opioid receptor in *T. canis* and leech ganglia as a positive control (Fig. 2). Using specific primers for μ-opioid receptor gene, we amplified the μ-opioid receptor from *T .canis* and leech ganglia as an expected size (441 bp), (Fig. 2).

## Discussion

 This study demonstrated the presence of morphine and its receptor expression under non-stimulated and *in vitro* condition in *T. canis* for the first time. All worms were kept five days before analysis and presence of morphine showed the ability of synthesis of this material by worm. Morphine is known as an immunosuppressive agent and this ability led to hypothesize of the secretion of morphine by parasitic worms may play a role in down-regulation of the host immune response.^18^^,^^19^ Administration of morphine in both animal and human decrease several natural and adaptive immunity function like cellular immunity.^20^ Modulation of immune response by morphine can be performed directly by binding to μ-opioid receptors on immune cell or by indirect pathway through binding to central nervous system receptors.^5^ Activation of indirect pathway cause production of immuno-suppressive agents like glucocorticoids and noradrenalin which act on leuko-cytes.^21^^,^^22^Also, opiate can activate sympathetic nervous system and via this route stimulate primary and secondary lymphoid organs (e.g. spleen) causing production of catecholamine which have been shown to suppress immune cells like macrophage, NK cells and lympho-cytes.^23^ Proliferation of T-lymphocyte and production of IL-2 and IFN-ϒ were shown to decrease following administration of opiate while type B lympho-cyte is less pronounced.^21^ Roy and colleagues expressed morphine inhibitory effect on monocyte/macrophage function and proliferation and differentiation of macrophage which cause decrease of phagocytosis of some pathogens.^24^

Presence of morphine like substance in invertebrates were documented in leech, *Mytilus edulis,*^18^^,^^25^ and *hirido medicinalis,*^7^ and support this idea that opiate may play a role in nervous and immune system and could inhibit cellular activation of immunocytes.^7^ In parasitic worms, morphine like molecule was first detected in *Schistosoma mansoni* by HPLC and raidoimmune assay (RIA),^26^ and by HPLC and mass spectrometry (MS).^27^ The secreted morphine cause immunocyte rounding and immobility, which is naloxone- reversible.^26^ In *Trichinella spiralis *presence of morphine like and codeine like molecule is shown by HPLC and confirmed by RIA.^28^ Zhu *et al*. described that the adult *Dracunclus medinensis* contained morphine and its active metabolite morphine-6-glucoronide.^27^ In pig intestinal nematode, *Ascaris suum*, presence of opiate alkaloid morphine was confirmed by HPLC coupled with gas chromatography–mass spectro-metry.^1^ They also showed the immuno-reactive morphine was found in sub-cuticle layers, hypoderms, and muscles underlying the skin and in the nerve chords by immuno-fluorescence assay.^1^

In the present study, we showed the expression of μ-opioid receptor in expected size of 441 bp. Presence of opioid receptors and their precursors are documented in free-living invertebrates.^14^^,^^29^ For example the presence of morphine and their μ-opioid receptors were shown in *Mytilus edulis* which these opiate is very complex and exhibited 95.0% sequence identify with human μ-opioid receptors.^27^^,^^28^ In contrast with our results, Goumon* et al*. could not express μ-opioid receptors in pig intestinal nematode, *Ascaris suum,* and hypothesized that this material act as an external signaling molecule to down regulate host immune system.^1^

From long time morphine is known as an immune suppressive agent.^6^ One of the mechanism of human to flush out toxins like parasitic worms is diarrhea. On the other hand, constipating effects of opium were concern and opium derivatives, used as an anti-diarrheal remedies beyond to analgesic properties.^30^

Thousand years ago Persian physician, Avicenna, prescribed morphine for cough, anemia and diarrhea.^31^ Based on these findings we hypothesized that morphine produced by *T. canis* can act on μ-opioid receptors on gastrointestinal lumen to diminish flushing out mechanism of host and cause constipation and they can live for long time in host body. The constipation is caused by morphine and other opiates mediated by stimulating non-propulsive motility pattern in gastrointestinal tract.^32^ Opiates inhibit neurotransmission in enteric ganglia and suppress inhibitory neural input to the muscle layers. Moreover, opiates can also inhibit the activity of intestinal secrete-motor neurons and cause inhibiting intestinal secretions.^32^

In conclusion, according to these findings, production of morphine like substances by parasite might be an important strategy to interact and communicate with host immune response. Meanwhile, further studies are needed to evaluate the morphine ability *in vivo *condition and in different developmental stages of *T. canis*.

## References

[1] Goumon Y, Casares F, Pryor S (2000). Ascaris suum, an intestinal parasite, produces morphine. J Immunol.

[2] Colley DG, LoVerde PT, Savioli L (2001). Medical helminthology in the 21st century. Science.

[3] Capron A, Dessaint JP, Capron M (1987). Immunity to schistosomes: Progress toward vaccine. Science.

[4] Pryor SC, Henry S, Sarfo J (2005). Endogenous morphine and parasitic helminthes. Med Sci Monit.

[5] Sacerdote P (2006). Opioids and the immune system. Palliat Med.

[6] Stefano GB, Scharrer B, Smith EM (1996). Opioid and opiate immunoregulatory processes. Crit Rev Immunol.

[7] Laurent V, Salzet B, Verger-Bocquet M (2000). Morphine-like substance in leech ganglia Evidence and immune modulation. Eur J Biochem.

[8] Sonetti D, Mola L, Casares F (1999). Endogenous morphine levels increase in molluscan neural and immune tissues after physical trauma. Brain Res.

[9] Soulsby EJL, Mönnig HO (1968). Helminths, arthropods, and protozoa of domesticated animals.

[10] Maizels RM (2013). Toxocara canis: Molecular basis of immune recognition and evasion. Vet Parasitol.

[11] Pawlowski Z (2001). Toxocariasis in humans: Clinical expression and treatment dilemma. J Helminthol.

[12] Armour J, Duncan JL, Dunn AM (1996). Veterinary Parasitology.

[13] Pinelli E, Aranzamendi C (2012). Toxocara infection and its association with allergic manifestations. Endocr Metab Immune Disord Drug Targets.

[14] Stefano GB, Salzet M (1999). Invertebrate opioid precursors: Evolutionary conservation and the significance of enzymatic processing. Int Rev Cytol.

[15] Bowman JW, Winterrowd CA, Friedman AR (1995). Nitric oxide mediates the inhibitory effects of SDPNFLRF-amide, a nematode FMRFamide-related neuropeptide, in Ascaris suum. J Neurophysiol.

[16] Mard SA, Neisi N, Solgi G (2012). Gastroprotective effect of NaHS against mucosal lesions induced by ischemia-reperfusion injury in rat. Dig Dis Sci.

[17] Mard SA, Veisi A, Ahangarpour A (2015). Gastric acid induces mucosal H2S release in rats by upregulating mRNA and protein expression of cystathionine gamma lyase. J Physiol Sci.

[18] Stefano GB, Scharrer B (1996). The presence of the mu3 opiate receptor in invertebrate neural tissues. Comp Biochem Physiol C Pharmacol Toxicol Endocrinol.

[19] Zhu W, Pryor SC, Putnam J (2004). Opiate alkaloids and nitric oxide production in the nematode Ascaris suum. J Parasitol.

[20] Sacerdote P, Bianchi M, Gaspani L (2000). The effects of tramadol and morphine on immune responses and pain after surgery in cancer patients. Anesth Analg.

[21] Budd K (2004). Pain, the immune system, and opioimmuno-toxicity. Rev Analg.

[22] Fecho K, Maslonek KA, Dykstra LA (1996). Assessment of the involvement of central nervous system and peripheral opioid receptors in the immunomodulatory effects of acute morphine treatment in rats. J Pharmacol Exp Ther.

[23] Flores LR, Hernandez MC, Bayer BM (1994). Acute immuno-suppressive effects of morphine: Lack of involvement of pituitary and adrenal factors. J Pharmacol Exp Ther.

[24] Roy S, Ramakrishnan S, Loh HH (1991). Chronic morphine treatment selectively suppresses macro-phage colony formation in bone marrow. Eur J Pharmacol.

[25] Stefano GB, Digenis A, Spector S (1993). Opiate-like substances in an invertebrate, an opiate receptor on invertebrate and human immunocytes, and a role in immunosuppression. Proc Natl Acad Sci USA.

[26] Leung MK, Dissous C, Capron A (1995). Schistosoma mansoni: The presence and potential use of opiate-like substances. Exp Parasitol.

[27] Zhu W, Baggerman G, Secor WE (2002). Dracunculus medinensis and Schistosoma mansoni contain opiate alkaloids. Ann Trop Med Parasitol.

[28] Pryor SC, Elizee R (2000). Evidence of opiates and opioid neuropeptides and their immune effects in parasitic invertebrates representing three different phyla: Schistosoma mansoni, Theromyzon tessulatum, Trichinella spiralis. Acta Biol Hung.

[29] Cadet P, Stefano GB (1999). Mytilus edulis pedal ganglia express mu opiate receptor transcripts exhibiting high sequence identity with human neuronal mu1. Brain Res Mol Brain Res.

[30] Bueno L, Fioramonti J (1988). Action of opiates on gastrointestinal function. Baillieres Clin Gastroenterol.

[31] Merlin MD (1984). On the trail of the ancient opium poppy.

[32] Wood JD, Galligan JJ (2004). Function of opioids in the enteric nervous system. Neurogastroenterol Motil.

